# Advances in Diagnostics and Management of Respiratory System Diseases

**DOI:** 10.3390/diagnostics14010020

**Published:** 2023-12-21

**Authors:** Paola Confalonieri, Francesco Salton, Marco Confalonieri, Riccardo Pozzan, Barbara Ruaro

**Affiliations:** 1 Pulmonology Unit, Department of Medical Surgical and Health Sciences, University of Trieste, Hospital of Cattinara, 34149 Trieste, Italy; 2 Pulmonology Unit, Azienda Ospedaliera Santa Maria degli Angeli, 33170 Pordenone, Italy

Recently, significant innovations in the diagnosis and management of respiratory diseases have been developed [1,2,3,4]. All aspects of respiratory medicine have seen significant progress in increasing the innovation and precision and reducing the invasiveness of all instruments and technologies used in the diagnosis and management of pulmonary diseases [1,2,3,4]. Firstly, more sophisticated and minimally invasive techniques have been developed in interventional pulmonology (i.e., endobronchial ultrasound, cryobiopsy and pleuroscopy) [1,2,3,4]. In addition to interventional techniques, significant advances in respiratory medicine in recent years have come from molecular medicine, thanks to the genotyping of respiratory diseases, such as lung cancer, which has provided diagnostic targets for more specific and personalised treatments [4,5]. Furthermore, by exploiting digital technologies, the application of telemedicine is likely to improve the management of respiratory diseases. In this special issue, three articles reported on interstitial lung disease, confirming that this is a hot topic in respiratory research [6,7,8]. In the first study, the authors conducted an observational, case–control, retrospective study to evaluate the role of the perinuclear anti-neutrophil cytoplasmic antibody (p-ANCA) in predicting clinical evolution and prognosis in patients with idiopathic pulmonary fibrosis (IPF) [6]. The researchers enrolled 18 IPF p-ANCA-positive patients and 36 IPF p-ANCA-negative patients. Half of the IPF patients with p-ANCA positivity were classified as microscopic polyangiitis (MPA). Interestingly, the paper showed that p-ANCA positivity, especially when associated with high levels of rheumatoid factor (RF), could predict the evolution of usual interstitial pneumonia (UIP), with a better prognosis than IPF. In conclusion, it is important to emphasise that ANCA testing should be included in the diagnostic routine of patients with UIP to help clinicians in the evaluation and follow-up of these complicated patients [6].

The second article is an interesting review in which the authors discuss the role, composition and timing of multidisciplinary diagnosis (MDD) with regard to IPF, interstitial lung disease (ILD), hypersensitivity pneumonitis and idiopathic pneumonia with autoimmune features, based on the most recent recommendations for the diagnosis of these complex diseases [7]. The authors emphasise that an MDD is essential to improve the accuracy of ILD diagnosis, enabling the optimisation of invasive procedures (e.g., transbronchial cryobiopsy) and improved therapy. In conclusion, the manuscript supported the fact that the MDD has been involved in achieving a continuous and dynamic process, often referred to as a ‘working diagnosis’, which involves the progressive integration and re-evaluation of clinical, radiological and histological features [7]. The aim of the third article was to perform a systematic review to evaluate the overall benefits of lung ultrasound (LUS) examination using high-resolution computed tomography (HRCT) as the gold standard for assessing the presence of interstitial lung disease (ILD) in patients with systemic sclerosis (SSc) [8]. The results of this meta-analysis indicated a high sensitivity and a low false-positive rate for most of the included papers regarding the role of LUS. In conclusion, the LUS examination proved to be a valuable tool for discerning which SSc patients should receive additional HRCT scans to detect ILD, thus reducing the doses of ionising radiation exposure in SSc patients [8]. The fourth article reported the case of a 35-year-old patient who developed a haemopneumothorax due to altitude barotrauma during an aeroplane flight, related to desquamative interstitial pneumonia (DIP) [9]. This article emphasised the importance of the CT findings and histopathological evaluation for the diagnosis of DIP. The aim of the last study was to assess whether the changes in the immune system detected by lymphocyte typing in peripheral blood correlated with the severity of sarcoidosis, evaluated according to two distinct severity scores proposed by Wasfi in 2006 and Hamzeh in 2010 [10]. In the eighty-one patients recruited, neither score showed an association with the level of total lymphocytes or lymphocyte subclasses. In conclusion, the lymphocyte subpopulation values at the time of diagnosis do not appear to correlate with the severity of the disease at onset [10].

In conclusion, the presentation of the latest research on lung diseases, especially ILD, has potential implications for increasing our knowledge in respiratory medicine and improving patient outcomes in the near future.
